# Endoscopic vs. microscopic stapes surgery: An anatomical feasibility study

**DOI:** 10.3389/fsurg.2022.1054342

**Published:** 2022-11-23

**Authors:** Esther E. Blijleven, Koen Willemsen, Ronald L. A. W. Bleys, Robert J. Stokroos, Inge Wegner, Henricus G. X. M. Thomeer

**Affiliations:** ^1^ Department of Otorhinolaryngology – Head and Neck Surgery, University Medical Center Utrecht, Utrecht, Netherlands; ^2^ Brain Center, University Medical Center Utrecht, Utrecht, Netherlands; ^3^ 3D Lab, Division of Surgical Specialties, University Medical Center Utrecht, Utrecht, Netherlands; ^4^ Department of Anatomy, University Medical Center Utrecht, Utrecht, Netherlands; ^5^ Department of Otorhinolaryngology – Head and Neck Surgery, University Medical Center Groningen, Groningen, Netherlands

**Keywords:** stapes surgery, otosclerosis, endoscopic approach, chorda tympani, minimally invasive

## Abstract

**Objectives:**

To investigate the feasibility of the endoscopic approach vs. microscopic approach during stapes surgery, focusing on the visualization of the important anatomical structures of the middle ear, the volume of the resected scutum and chorda tympani (CT) injury.

**Methods:**

Fresh frozen human cadaveric heads underwent two stapes surgeries using an operating microscope on one ear and an endoscope on the other ear. The surgeon documented the visualization of critical landmarks, as well as exposure and injury of the CT. The volume of resected scutum was evaluated using cone beam computed tomography scanning and three-dimensional imaging.

**Results:**

We performed endoscopic stapes surgery in 10 ears and microscopic stapes surgery in 11 ears. A stapes prosthesis was placed in all ears. The volume of bony scutum resection was significantly lower in the endoscopic group (median = 2.20 mm^3^, IQR = 4.17) than in the microscopic group (median 13.25 mm^3^, IQR = 8.71). No scutum was removed in two endoscopic ears, while scutum was removed in all microscopic ears. The endoscopic and microscopic group had similar CT injury.

**Conclusions:**

This study showed that the endoscopic stapes surgery procedure is feasible and might be less invasive than microscopic stapes surgery. Future clinical prospective and functional studies will be needed to support our findings.

## Introduction

In recent years, the use of an endoscope in stapes surgery has increased because of the associated improved visualization of the anatomical structures of the middle ear. The microscope only provides a straight-line view, while the endoscope offers a panoramic visualization of the middle ear anatomy and a more detailed view of the structures (1–5). Using an endoscope may therefore be advantageous in patients with anatomical abnormalities of the middle ear, such as stapes malformations (6). Additionally, a better visualization of the middle ear may result in less removal of the bony wall of the most medial segment of the external acoustic meatus, also known as the scutum (7, 8). Removal of scutum can lead to exposure, manipulation and injury of the chorda tympani (CT), resulting in postoperative dysgeusia. If using an endoscope results in more anatomical structure preservation (i.e., scutum left intact), it may also lead to less postoperative dysgeusia (2–4, 9).

The use of an endoscope during stapes surgery is not widespread for multiple reasons. First of all using an endoscope could limit depth perception, which might interfere with safe footplate fenestration, especially in case of a deep oval window niche (1, 2). Secondly, there may be a longer learning curve to get used to the single-handed technique. However, the use of an endoscope holder can enable the two-handed technique for stapes surgery (10).

This study aims to investigate the feasibility of the endoscopic approach vs. microscopic approach during stapes surgery, focusing on the visualization of the important anatomical structures of the middle ear, the volume of the resected scutum and CT injury.

## Materials and methods

### Specimens

In a tertiary referral center, an anatomical feasibility study was performed with fresh frozen human cadaveric heads. The specimens were derived from bodies that were donated to the department of Anatomy of the University Medical Center Utrecht. In this donation program, people offer their entire bodies without using a tissue bank intermediary. From these persons, written informed consent was obtained during life that allowed the use of their entire bodies for education and research purposes. The possibility for body donation is part of the Dutch law on dead bodies.

An ear was excluded if the temporal bone showed signs of trauma, previous middle ear surgery or anatomical abnormalities of the middle ear on the preoperative cone beam computed tomography (CBCT) scan or during surgery. Some specimens underwent surgery on one ear, because the other ear was excluded based on the exclusion criteria. Therefore, we refer to ears instead of specimens throughout this article.

The authors confirm that all procedures during this study are in accordance with the relevant national and international guidelines for human experimentation. No approval of the Institutional Review Board was required for this anatomical study.

### Surgical technique and intraoperative parameters

The cadaveric heads were thawed for at least 24 h. Each specimen underwent two stapes surgeries using a microscope on one ear and an endoscope on the other ear. Alternately, the endoscopic and the microscopic approach were used for the left and the right ear. All surgeries were performed by one ENT surgeon (H.G.X.M.T), who used an endoscope with an outer diameter of 4 mm, a length of 18 cm and a 30° angle (Olympus, Leiderdorp, the Netherlands). The ENT surgeon had four years of experience with endoscopic middle ear surgeries.

All stapes surgeries were carried out as follows: an endaural procedure with an intercartilaginous incision of 2 cm–3 cm was performed, followed by a Rosen's incision and dissection of a tympanomeatal flap. When necessary, the surgeon resected the scutum with a curette in the endoscopic ears and an osteotome and curette in the microscopic ears to view the middle ear landmarks. Middle ear landmarks were preoperatively determined for optimal exposition during stapes surgery to perform a safe procedure. These are the oval window niche (including facial nerve, long process of the incus and pyramidal eminence), entire stapes footplate, stapes superstructure and stapedius muscle. When necessary, the CT was manipulated (handled, stretched or sectioned) during the visualization process of the middle ear. For example, if the CT was partly transected, it was coded as stretched. The stapedius muscle and the posterior crus of the stapes superstructure were cut and the anterior crus of the stapes superstructure was fractured and removed. The stapes footplate was fenestrated with a micropick and a Schuknecht-type Teflon wire prosthesis (Medtronic Xomed, Jacksonville, FL, USA) was placed in the fenestration. In order to analyze the visualization of the anatomical structures of the middle ear, several pictures were taken of the endoscopic and microscopic view during the surgeries. Postoperatively, the surgeon (H.G.X.M.T) also documented the visualization of the middle ear landmarks and the exposure and injury of the CT.

### CBCT scan and analysis

**Figure 1:**
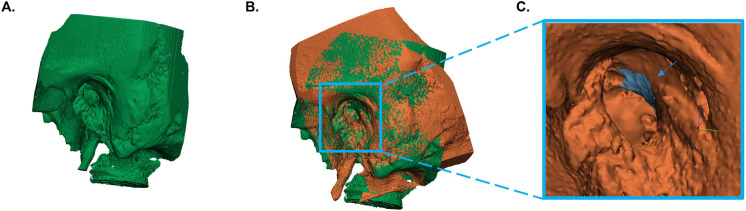
Volumetric three-dimensional (3D) evaluation of scutum removal. (**A**) Segmentation and conversion of the preoperative temporal bone images into a 3D model. (**B**) Alignment of the preoperative and postoperative 3D model. (**C**) Calculation of the bone volume of the resected scutum. The blue area represents the volume of resected scutum (see blue arrow). The orange temporal bone is the postoperative model.

The acquisition of temporal bone images was done preoperatively and postoperatively in all included specimens with a CBCT (VGi evo, NewTom, Cefla C.S., Italy) to assess the differences in the volume of resected scutum between the microscopic and endoscopic approach. The field of view (FoV) was set to 5 × 5 cm and the slice thickness to 0.3 mm in both scans. Images were stored in Digital Imaging and Communications in Medicine (DICOM) format and exported to Mimics version 21.0 (Materialise NV, Leuven, Belgium). The bony structures of the preoperative and postoperative temporal bone images were semi-automatically segmented from surrounding tissues using a threshold of 500 Hounsfield units and converted into a three-dimensional (3D) STL model in Mimics (11). The temporomandibular joint and ossicles were removed from the preoperative and postoperative 3D model, because the exact position of these anatomical structures may vary from (preoperative) scan to (postoperative) scan. Afterwards, the 3D models were imported into 3-matic medical design software version 13.0 (Materialise NV, Leuven, Belgium) for image fusion of the preoperative and postoperative 3D model for each ear to ensure that the scutum of both 3D models was in the same position. Ten identical landmarks were identified in both 3D models, which guided and tracked the image alignment of the preoperative and postoperative model in each ear. The final version of image alignment was visually verified by the researcher (E.E.B.). A 3D model of the bone volume of the resected scutum was calculated by subtracting the postoperative 3D model from the preoperative 3D model. All volume measurements were recorded in cubic millimeters. Figure 1 shows the steps used for the volumetric 3D evaluation in a microscopic ear.

**Figure 2:**
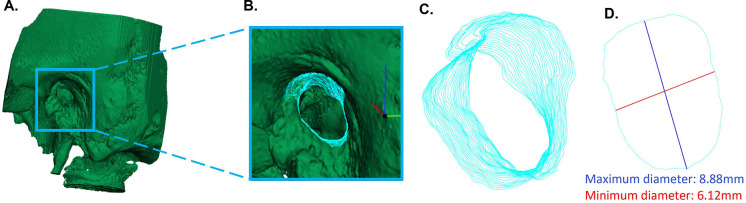
Measurement of the minimum and maximum diameter of the osseous external auditory canal (OEAC). (**A**) Segmentation and conversion of the preoperative temporal bone images into a three-dimensional (3D) model. (**B**) Detection of the elliptical shape of each 0.3 slice of the OEAC in the preoperative 3D model. (**C**) Crossections of the OEAC perpendicular to the viewing direction of the otorhinolaryngologist. (**D**) The maximum diameter (blue line) and minimum diameter (red line) of one slice of the OEAC.

3-matic was also used to measure the minimum and maximum diameter of the osseous external auditory canal (OEAC) in the preoperative 3D model. We selected the OEAC in the preoperative model and the software calculated the elliptical shape of each 0.3 mm slice of the OEAC. These cross-sections were perpendicular to the viewing direction during the stapes surgery. The centroid of the elliptical shape was detected and the minimum and maximum diameter for each OEAC sagittal slice were measured. The smallest minimum and largest maximum of all slices were determined as the minimum and maximum diameter of the ear. To increase the reproducibility, all above mentioned steps measuring the diameters, were scripted in Python. Figure 2 shows the steps used for the measurement of the minimum and maximum diameter of the OEAC. Ears were excluded from measuring diameters if the OEAC was not completely captured on CBCT scan images. All analyses with the 3D-imaging software were performed by the same author (E.E.B). We calculated correlations between the minimum and maximum diameter of the OEAC, smallest minimum and smallest maximum diameter of the OEAC and the volume of the resected scutum and the minimum diameter of the OEAC.

### Statistical analysis

Median and interquartile ranges (IQRs) were calculated for continuous variables. Continuous variables were tested for normality. A normal distribution of the data was assumed when the median was more or less equal to the mean, the standard deviation could be subtracted twice from the mean and histograms followed the normal distribution. A normal distribution of the data could not be assumed. Therefore, the nonparametric Mann-Whitney U test was used to analyze the differences in continuous variables between the microscopic and the endoscopic group. The Spearman's rank correlation coefficient was used to analyze the differences between the continuous variables. Categorical variables were summarized by frequency and percentage. The statistical analyses were performed using IBM SPSS Statistics version 27.0 (IBM Corp., Armonk, NY, USA).

## Results

We scanned 22 fresh frozen ears. One ear was excluded because it had undergone middle ear surgery during life. Therefore, we performed endoscopic stapes surgery in 10 ears and microscopic stapes surgery in 11 ears.

**Figure 3:**
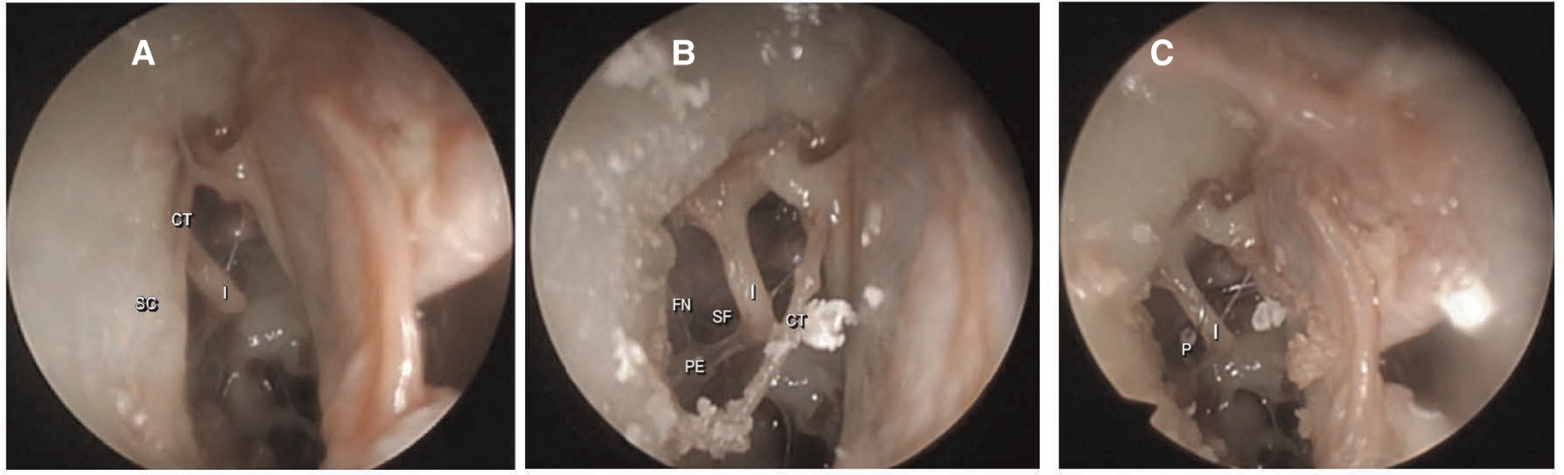
Endoscopic photographic view of the middle ear landmarks using a 4 mm endoscope with a 30° angle. (**A**) After dissection of the tympanomeatal flap; CT (chorda tympani), I (long process of the incus) and SC (scutum). (**B**) After scutum removal; PE (pyramidal eminence), FN (facial nerve) and SF (stapes footplate). (**C**) After prosthesis placement; *P* (prosthesis).

When we analyzed the surgical endoscopic view, all 10 ears had a complete intraoperative exposure of the middle ear landmarks. Figure 3 shows photographic images of the endoscopic visualization of the landmarks during surgery in the same ear. In all ears, a stapes prosthesis was placed directly in the fenestration of the stapes footplate. No endoscopic-induced lesions occurred during the surgeries.

In the microscopic ears, the long process of the incus was generally less visible after dissection of the tympanomeatal flap. In most microscopic ears the anterior crus of the stapes superstructure was not visible, so a blind fracturing procedure of this crus was done in these cases. The other landmarks of the middle ear were readily visible during piston placement.

**Figure 4:**
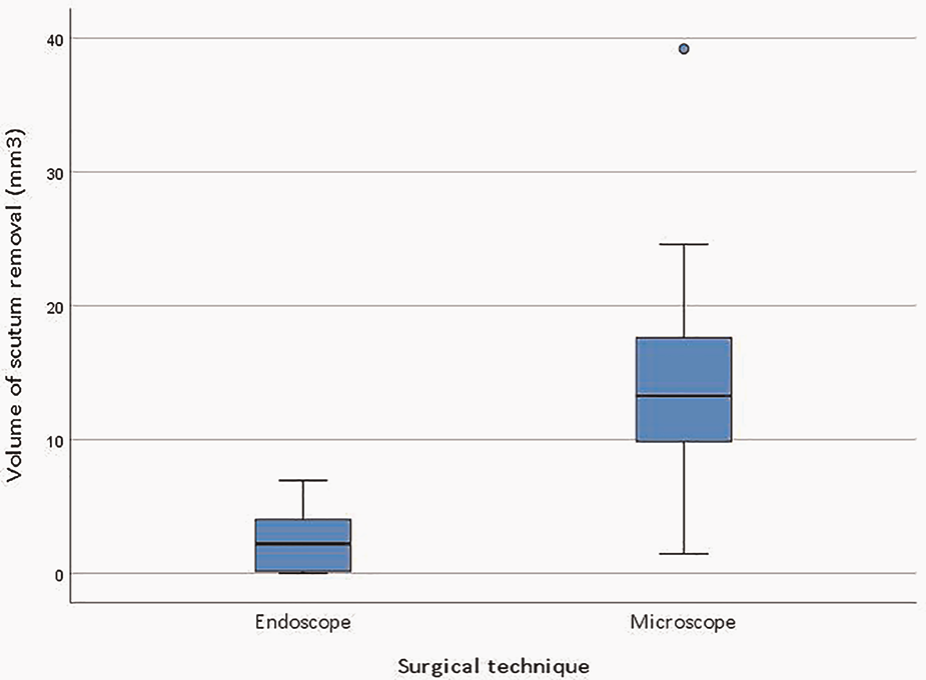
Boxplots of the resected scutum volume for the endoscopic (*n* = 10) and microscopic (*n* = 11) group. The Mann-Whitney *U* test was used to assess the difference in scutum resection between the two groups (*p* = 0.001).

**Figure 5:**
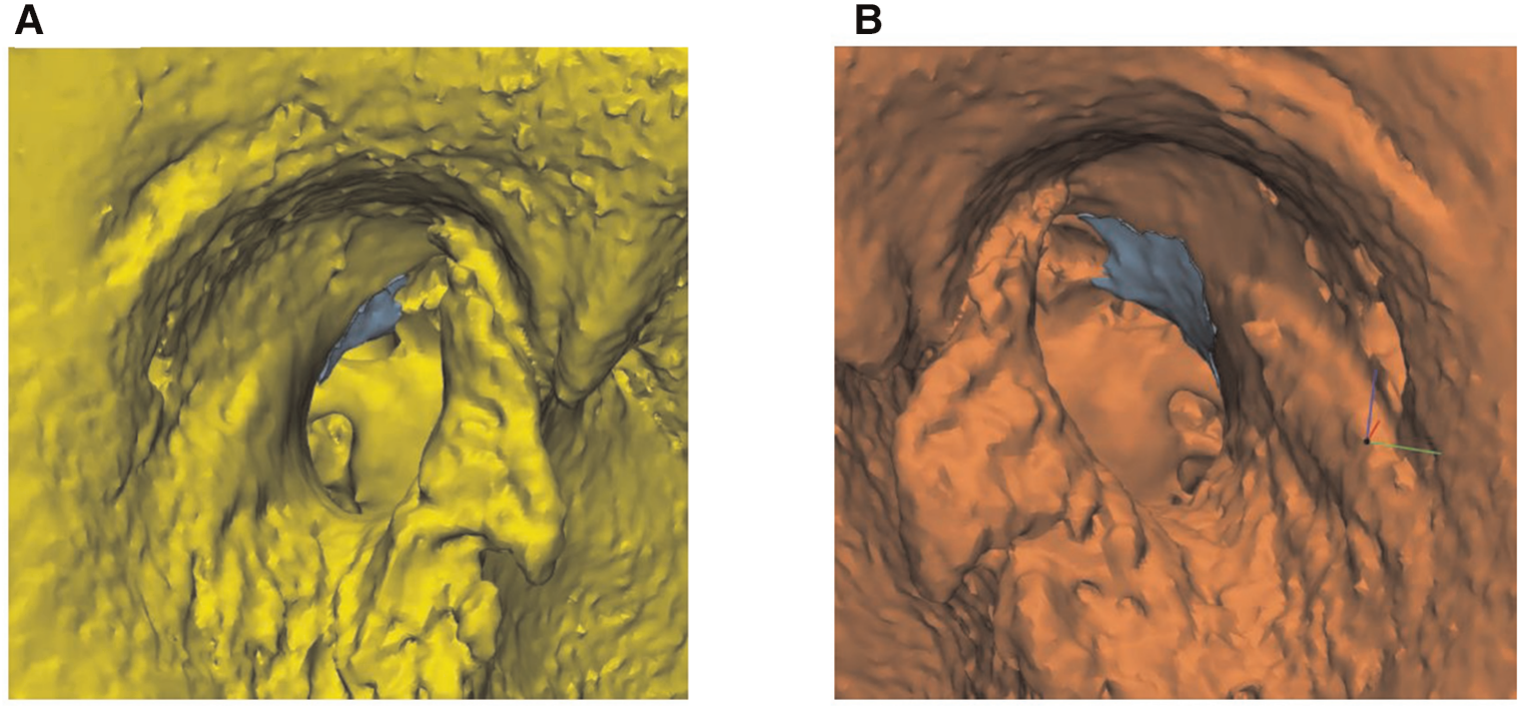
Volumetric three-dimensional (3D) evaluation of scutum removal in an endoscopic and microscopic ear. (**A**) The blue area represents the resected scutum volume in an endoscopic ear. (**B**) The blue area represents the resected scutum volume in a microscopic ear.

The median volume of resected scutum was 2.20 mm^3^ (IQR = 4.17) for the endoscopic ears and 13.25 mm^3^ (IQR = 8.71) for the microscopic ears. Figure 4 shows boxplots of the resected scutum volume for the endoscopic and microscopic groups. The difference in resected scutum volume was statistically significant (*p* = 0.001) between the endoscopic and microscopic ears. Scutum was not removed in two endoscopic ears because the middle ear landmarks were clearly visible after dissection of the tympanomeatal flap. Part of the scutum was removed in all microscopic ears. Figure 5 shows the resected scutum volume in a microscopic and an endoscopic ear.

One ear out of the 21 ears was excluded for the measurement of the diameters because the OAEC was not completely captured on the CBCT scan. The median maximum diameter of the OEAC was 10.79 mm (IQR = 2.36). The median minimum diameter of the OEAC was 5.45 mm (IQR = 1.67). The minimum and maximum diameter of the OEAC did not differ statistically significant between the microscopic and the endoscopic group. We found no correlation between the volume of resected scutum and the minimum and maximum diameter of the OEAC. We found no statistically significant correlation between the minimum and maximum diameter of the OEAC. However, we found a statistically significant moderate correlation of 0.52 (*p* = 0.02) between the smallest minimum diameter and the smallest maximum diameter along the entire length of the OEAC.

Table 1 shows that the CT was preserved in four endoscopic ears (40%) and in two microscopic ears (18%). The CT was sectioned in one ear in both groups. There was no statistically significant difference in CT trauma between the endoscopic and the microscopic ears.

**Table 1:** Chorda tympani (CT) trauma by surgical technique. There was no statistically significant difference in CT trauma between the endoscopic and the microscopic approach.

No. of Ears (%)		
	Endoscope	Microscope
CTN preserved	4 (40)	2 (18)
CTN handled	2 (20)	6 (55)
CTN stretched	3 (30)	2 (18)
CTN sectioned	1 (10)	1 (9)

## Discussion

### Literature overview

We found that endoscopic stapes surgery is feasible and that the endoscope allows the surgeon to visualize middle ear landmarks more in detail than the microscope, which is in line with previous findings (2, 9). However, in our study, the CT trauma was similar in both surgeries, unlike other research reports (4, 9). One reason for this difference could be the small sample size. Previously published studies included at least double the number of ears. Although there is no difference in CT trauma, in two endoscopic ears the scutum was left intact, whereas in all of the microscopic ears scutum was resected. Moreover, a larger proportion of scutum had to be removed using the microscopic approach than the endoscopic approach. No scutum resection reduces the likelihood of, CT manipulation and postoperative dysgeusia (12). Also, less invasive surgery might lead to less scar formation and complaints during the first weeks of the postoperative course. Especially given the trend towards more day case in stapes surgery, minimally invasive surgery goes hand in hand (13).

To the best of our knowledge, volumetric 3D measurement of scutum resection has never before been performed in cadaveric heads undergoing stapes surgery. Several studies reported that the endoscopic approach resulted in less scutum resection, but none of these studies used volumetric measurements of scutum resection (9, 14). Volumetric measurements of scutum resection have only been used to assess the invasiveness of microscopic and endoscopic cholesteatoma surgery. However, our results are incomparable with cholesteatoma research (15), because during cholesteatoma surgery, other middle ear landmarks must be visualized, such as the sinus tympani, which leads to a larger resected scutum volume.

**Figure 6:**
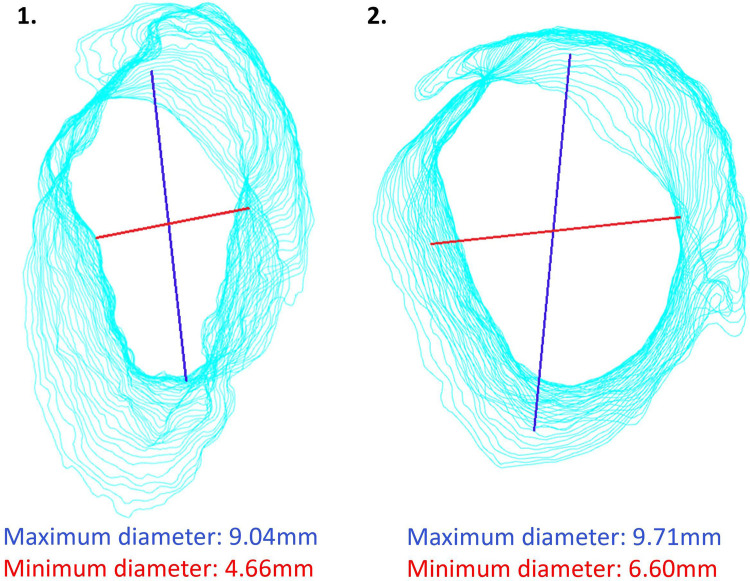
Two different ear canal characteristics. The blue line is the maximum diameter and the red line is the minimum diameter.

We measured the minimum and maximum diameter of the OEAC, because it is interesting to know from a surgical perspective whether the shape and diameter of the bony ear canal is of predictive value for successful stapes surgery. For the measurements of the diameter of the OEAC, we used the centroid of the OEAC's elliptical shape rather than the Feret diameter (see Figure 2) (16, 17). Using the Feret diameter would be incorrect in our study, as it leads to the largest minimum diameter rather than the smallest minimum diameter of the elliptical shape of each 0.3 slice of the OEAC. The significant moderate correlation between the smallest minimum and maximum diameter of the OEAC were in line with the literature (16). It might be an interesting additional surgical aid to be acquainted with the bony diameter along the ear canal (from lateral to medial) and to visualize its spatial orientation (and 3D direction). The visibility of the stapes might be limited when the angle of the OEAC is 30° or more in relation to the horizontal plane (16). See as an example two different ear canal characteristics in Figure 6. We could use these measurements of the diameter of the OEAC to make a preoperative surgical guide that predicts the feasibility of the endoscopic approach for each patient (16), which could be helpful in patients with congenital ear deformities. Future prospective *in vivo* studies will be necessary to further elaborate on this strategy and support its usefulness.

### Clinical use endoscope

In literature it is stated that an endoscope with a diameter of 3 mm and a length of 14 cm is the best endoscope to perform endoscopic stapes surgery (2). However, an endoscope with a diameter of 4 mm and a length of 18 cm is readily available in most hospitals, as this endoscope is often used for functional endoscopic sinus surgeries. A 3 mm endoscope is known to realize minimal middle ear temperature changes during surgery (2, 18). Higher temperature changes have been associated with larger diameters of endoscope (2, 19). No visible endoscopic-induced lesions, i.e., thermal injury, were encountered during our surgeries. However, the cadaveric heads were of lower temperature than a body temperature. Using a LED source with 50% power and intermittent rinsing can also prevent temperature changes (20). The advantage of the 4 mm endoscope is the superior view (brighter exposure of anatomical structures and improved visualization). The advantage of a longer endoscope is better image stabilization. A shorter endoscope might interfere with surgical manipulation or block movements of the other hand. To overcome this issue, a 30° angle of view and shorter instruments help in this case, because both hands remain in different horizontal planes (2, 21, 22). An endoscope with a 30° angle also provides a better visualization of the surgical field compared to the endoscope with a 0° angle. New endoscopes with improved lighting and optical visual qualities are the focus of the companies providing these tools. It is questionable and reason for further research whether the applied tools are suboptimal compared to the suggested scope (14 cm length respectively 3 mm diameter).

The main disadvantage of the endoscope is believed to be the single-handed surgery in areas where even minimal bleeding compromises view and regular aspiration is then needed. Therefore, preoperative preparation is key: slow and adequate infiltration (lidocaine 1%/epinephrine 1:200.000) of 4 quadrants of the ear canal, application of epinephrine patties (1:1000 concentration) in the ear canal (vasoconstriction). Indeed, because two handed surgery is the mainstay in regular otologic surgery, a learning curve for this technique is expected and therefore in the beginning a longer operating time. However, literature showed that after 10 endoscopic stapes surgeries for an ENT surgeon experienced in microscopic stapes surgery, there was no statistically significant difference in operating time compared to the microscopic stapes surgeries nor in the amount of postoperative adverse events (23). Also, based on postoperative audiometric results, the endoscopic and the microscopic approach have similar surgical success (7, 9, 12, 23–26). Finally, the use of an endoscope holder can facilitate the two-handed technique of stapes surgery and reduce the fatigue associated with holding the endoscope for extended periods of time (10). Therefore, we believe that the endoscopic approach in stapes surgery deserves a place in the surgical armamentarium given its additional values. Provided that the learning curve of an inexperienced ENT surgeon will be even longer, one might preserve this innovation only for the experienced surgeon (12, 23, 25, 26).

Finally, using the endoscope provides more optimal circumstances than the microscope in educational settings and for training purposes. Residents identified the middle ear landmarks more easily and understood the surgical steps and technique better when using an endoscope (23, 27). Lastly, supervising a trainee is more insightful during endoscopic stapes surgery, as monocular microscopic view (or through screen) significantly differs between the operating trainee and the supervisor during stapes surgery. During endoscopic stapes surgery, the trainee and the supervisor have the same view.

### Strengths and limitations

Our study had different strengths. First of all, we minimized intra- and inter-observer variability. One experienced ENT surgeon performed all stapes surgery procedures and documented the visualization of the middle ear landmarks and the exposure as well as injury of the CT. Secondly, this is the first study visualizing anatomical differences pre- and postoperatively, using 3D models, providing more insight in the usefulness of endoscopic surgery. Thirdly, this study provides insight in a possible relationship between ear canal shape and surgical feasibility. A limitation of the study is the small sample size leading to weak methodological conclusions. Also, the tissue handling in fresh frozen cadaveric temporal bones is different than *in vivo* although closest to real life situation. Therefore, conclusions drawn should be verified in future clinical settings to support our findings.

## Conclusions

The endoscopic stapes surgery procedure is feasible in experienced hands and might potentially be less invasive than the microscopic procedure. In educational centers, we recommend the use of an endoscope. Future prospective and functional studies will be needed to support our findings.

## Data Availability

The raw data supporting the conclusions of this article will be made available by the authors, without undue reservation.
